# Microglial P2X4 receptors are essential for spinal neurons hyperexcitability and tactile allodynia in male and female neuropathic mice

**DOI:** 10.1016/j.isci.2023.108110

**Published:** 2023-10-02

**Authors:** Damien Gilabert, Alexia Duveau, Sara Carracedo, Nathalie Linck, Adeline Langla, Rieko Muramatsu, Friedrich Koch-Nolte, François Rassendren, Thomas Grutter, Pascal Fossat, Eric Boué-Grabot, Lauriane Ulmann

**Affiliations:** 1 IGF, University Montpellier, CNRS, INSERM, F-34094 Montpellier, France; 2 LabEx Ion Channel Science and Therapeutics, Montpellier, France; 3 University Bordeaux, CNRS, IMN, UMR 5293, F-33000 Bordeaux, France; 4 Department of Molecular Pharmacology, National Institute of Neuroscience, National Center of Neurology and Psychiatry, Tokyo 187-8502, Japan; 5 Institute of Immunology, University Medical Center Hamburg-Eppendorf, D-20246 Hamburg, Germany; 6 University of Strasbourg, CNRS, CAMB UMR 7199, F-67000 Strasbourg, France

**Keywords:** Natural sciences, Biological sciences, Physiology, Pathophysiology, Neuroscience, Behavioral neuroscience, Sensory neuroscience

## Abstract

In neuropathic pain, recent evidence has highlighted a sex-dependent role of the P2X4 receptor in spinal microglia in the development of tactile allodynia following nerve injury. Here, using internalization-defective P2X4mCherryIN knockin mice (P2X4KI), we demonstrate that increased cell surface expression of P2X4 induces hypersensitivity to mechanical stimulations and hyperexcitability in spinal cord neurons of both male and female naive mice. During neuropathy, both wild-type (WT) and P2X4KI mice of both sexes develop tactile allodynia accompanied by spinal neuron hyperexcitability. These responses are selectively associated with P2X4, as they are absent in global P2X4KO or myeloid-specific P2X4KO mice. We show that P2X4 is *de novo* expressed in reactive microglia in neuropathic WT and P2X4KI mice of both sexes and that tactile allodynia is relieved by pharmacological blockade of P2X4 or TrkB. These results show that the upregulation of P2X4 in microglia is crucial for neuropathic pain, regardless of sex.

## Highlights

• Increased cell surface P2X4 induces hypersensitivity in naive mice of both sexes
• P2X4 is *de novo* expressed in reactive microglia in neuropathic mice of both sexes
• Microglial P2X4 is crucial for spinal neuronal hyperexcitability regardless of sex
• Microglial P2X4 is crucial for neuropathic pain regardless of sex

## Graphical abstract

**Figure:**
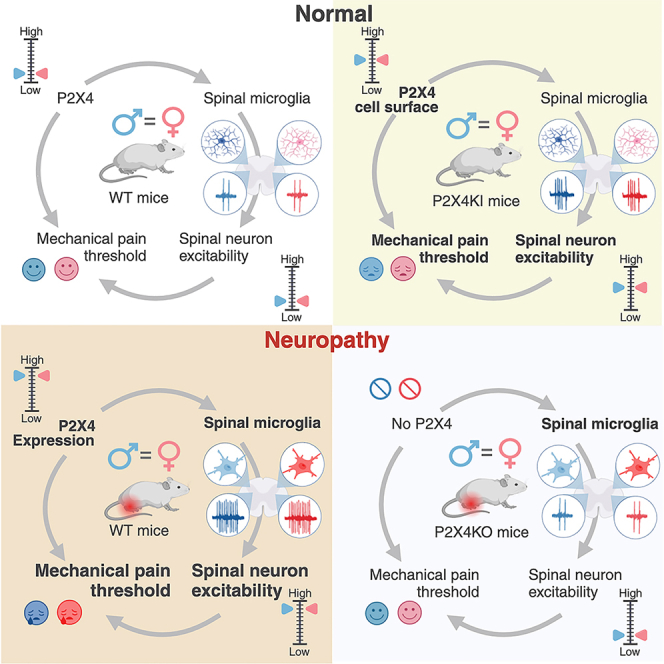

## Introduction

It is estimated that 10% of the worldwide population suffers from chronic neuropathic pain.^1^ Therapeutic strategies currently developed are mainly based on opioids leading to the development of side effects, dependency, paradoxical hyperalgesia, and early death.^2^ The lack of efficient treatments has resulted in the development of psychiatric comorbidities such as depression, sleeping disorders, or opioids addiction that strengthen marginalization and the drastic decrease of the patient’s quality of life.^3^^,^^4^ Neuropathic pain is primarily associated with a nerve lesion, and is characterized by microglia reactivity and central sensitization, pointing out the establishment of neuroimmune system interaction that might play crucial roles in the development and the maintenance of pathological pain. At the cellular level, chronic neuropathic pain syndromes, such as mechanical allodynia and hyperalgesia, are associated with strong microglia reactivity in the ipsilateral side of the damaged dorsal horn of the spinal cord.^5^ Reactive states of microglia include morphological hypertrophy, proliferation, and expression of various genes such as the purinergic ATP-gated P2X4 receptors (P2X4).^5^^,^^6^ P2X4 expression is strongly increased in reactive microglia^5^ and their activation contributes to the spinal inflammatory responses and functional remodeling of local synaptic networks.^6^ In males, activation of upregulated P2X4 in spinal microglia by *de novo* expression leads to the release of brain-derived neurotrophic factor (BDNF) which, via activation of its neuronal TrkB receptor, induces GABAergic disinhibition through the neuronal decrease of the chloride gradient triggers by the downregulation of the K^+^/Cl^−^ potassium-chloride transporter member 2 (KCC2) co-transporter.^7^^,^^8^ A consequence of this disinhibition is the development of a hyperexcitability of local spinal network, underlying allodynia, a hallmark of neuropathic pain.^7^ On the other hand, recent studies suggest that a divergent P2X4-independent pathway is developed in females.^9^ Several convincing data indicate that contrary to males, p2rx4 mRNA and P2X4 protein are not upregulated in female spinal microglia after neuropathy and that tactile allodynia was achieved through a mechanism that involves lymphocytes but not microglia P2X4.^9^^,^^10^ Interestingly, despite these differences, both male and female mice with neuropathic pain exhibit a convergent downregulation of KCC2 and subsequent development of mechanical hypersensitivity.^11^ Other studies have indicated that microglial P2X4 and BDNF/TrkB signaling contribute to bone cancer^12^ and herpetic pain^13^ models in female rodents. These findings emphasize the complexity of the dialogue between microglia and neurons in the spinal network and demonstrate the need for additional studies to clarify this sexual dimorphism chronic pain.

In addition to the microglial expression, neuronal expression of P2X4 in both sexes during painful conditions (i.e., peripheral inflammation) has been documented in sensory neurons^14^ or in different brain structures where they are associated with synaptic plasticity.^15^^,^^16^^,^^17^ Increased expression of neuronal P2X4 has also been observed in mouse models of neurodegenerative diseases, such as Alzheimer’s disease or amyotrophic lateral sclerosis (ALS) (for review, see the study by Duveau et al. and Montilla et al,^18^^,^^19^). Using internalization-defective P2X4mCherryIN knockin mice (P2X4KI mice), it has recently been shown that increased surface P2X4 in hippocampal neurons reduces anxiety and impairs memory,^20^ while increased P2X4 in both neurons and microglia is instrumental for ALS pathogenesis.^21^ This suggests that neuronal P2X4 could, in parallel or with opposite effects of microglia, take part of cellular network modifications or sex differences observed according to the studied model. It was thus important to determine the cell- and sex-specific involvement of P2X4 in neuropathic pain.

In the present study, we unraveled the role of P2X4 in neuropathic pain and hyperexcitability of local spinal network using several pharmacological and genetic tools invalidating P2X4 expression either in the whole body (P2X4 knockout (P2X4KO) mice) or selectively in myeloid cells P2X4-deleted mice (myeloid P2X4KO mice) and increasing cell membrane P2X4 (P2X4KI mice).^20^ Overall, our results show that cell surface P2X4 is sufficient to induce mechanical hypersensitivity in mice of both sexes and that the upregulation of P2X4 in microglia following spared nerve injury (SNI) is crucial for neuropathic pain and spinal neuronal hyperexcitability regardless of sex.

## Results

### Increased P2X4 is sufficient to induce mechanical hypersensitivity and necessary for neuropathic pain in both male and female mice

**Figure 1:**
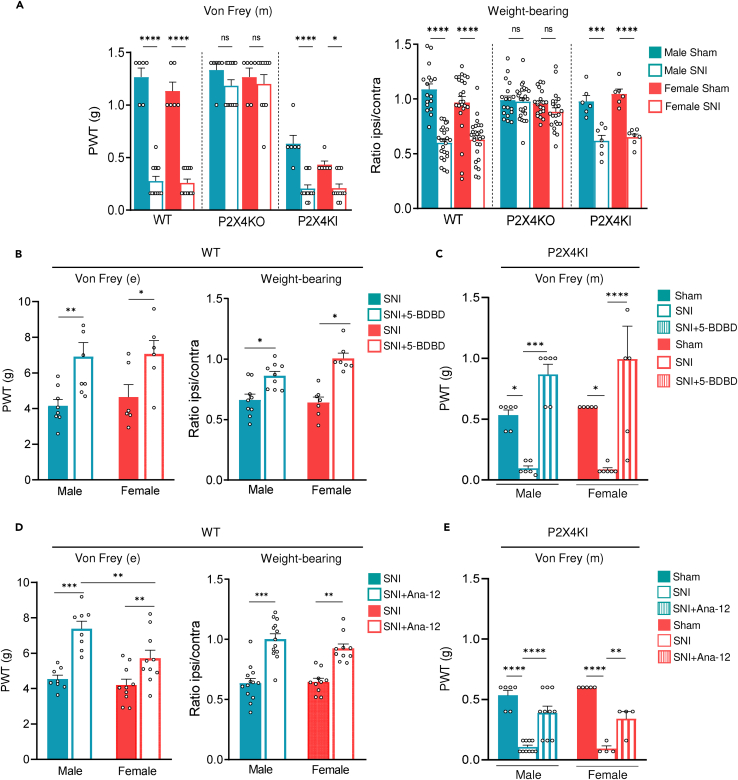
Surface P2X4 is involved in mechanical neuropathic pain in males and females (A) Manual Von Frey (Von Frey (m), left panel) and weight-bearing tests (right panel) for the assessment of mechanical sensitivity of sham and SNI male and female WT, P2X4KO, and P2X4KI mice 15 days after surgery. SNI induces a significant decrease of the mechanical paw withdrawal threshold (PWT) in both male and female WT and P2X4KI mice, while P2X4KO mice do not develop mechanical hypersensitivity. Note the basal mechanical hypersensitivity in P2X4KI mice. n = 6–12 mice per condition. ∗∗∗∗p < 0.0005, unpaired Student’s *t* test. Using the weight-bearing test, the ipsilateral/contralateral ratio is decreased in WT and P2X4KI mice, but is unchanged in P2X4KO mice. Data are represented as mean ± SEM. N = 17–24 mice per condition. ∗∗∗∗p < 0.0005, Three-way ANOVA. (B) Mechanical sensitivity measured with the electronic Von Frey (Von Frey (e)) 2 h after the P2X4 antagonist 5-BDBD (28 mg/kg) i.p. injection in WT and P2X4KI SNI mice (15 days post surgery). 5-BDBD reverses mechanical sensitivity in male and female mice. Data are represented as mean ± SEM. N = 7–9 mice. ∗p < 0.05, ∗∗p < 0.01, ∗∗∗p < 0.001, ∗∗∗∗p < 0.005, two-way ANOVA. (C) Von Frey test measurements from P2X4KI male and female mice in sham, SNI, and SNI + 5-BDBD injection. Data are represented as mean ± SEM. N = 6 mice per condition. ∗p < 0.05, ∗∗p < 0.01, ∗∗∗p < 0.001, ∗∗∗∗p < 0.005, two-way ANOVA. (D) Mechanical sensitivity 2 h after the TrkB antagonist Ana-12 (1 mg/kg) i.p. injection in WT SNI mice (15 days post surgery). Ana-12 reverses mechanical hypersensitivity in male and female mice. Data are represented as mean ± SEM. N = 8–10 mice, ∗∗p < 0.01, ∗∗∗p < 0.001, two-way ANOVA. (E) Von Frey test measurements from P2X4KI male and female mice in sham, SNI, and SNI + Ana-12 injection. Data are represented as mean ± SEM. N = 6 mice per condition. ∗p < 0.05, ∗∗p < 0.01, ∗∗∗p < 0.001, ∗∗∗∗p < 0.005, two-way ANOVA. See also Figure S1.

To determine the extent to which increased surface expression of P2X4 contributes to mechanical hypersensitivity in both male and female mice, we first took advantage of internalization-defective P2X4KI mice which exhibit enhanced cell surface P2X4 expression in all cells naturally expressing P2X4.^20^ We first compared the mechanical sensitivity of naive wild-type (WT), P2X4KI, and P2X4KO mice of both sexes using manual Von Frey test. Interestingly, P2X4KI mice displayed a significant increase in mechanical sensitivity compared to WT or P2X4KO mice, suggesting that P2X4KI mice exhibited hypersensitivity (Figures 1A, left panel and S1A). In addition, this hypersensitivity was similar in both male and female P2X4KI mice, suggesting that increased cell surface P2X4 is sufficient to trigger mechanical hypersensitivity in mice, regardless of sex.

We then compared the pain sensitivity induced by SNI in male and female WT, P2X4KI, and P2X4KO mice. As shown in Figure 1A (left panel), 15 days after neuropathy, significant decreases in the ipsilateral threshold were detected in both male and female WT mice using manual Von Frey test. In P2X4KI mice and despite the reduced basal threshold, SNI performed on these mice induced also a significant reduction compared to the basal threshold in both male and female mice. The extent of tactile allodynia developed from day 3, and extended at least 21 days after surgery was similar for WT and P2X4KI mice of both sexes (Figure S1B). By contrast and consistent with previous data,^22^ the threshold of mechanical sensitivity remained unchanged by SNI procedure in both P2X4KO male and P2X4KO female mice. These results showed that the deletion of P2X4 was sufficient to suppress SNI-induced tactile allodynia, irrespective of sex. To extend this result to other neuropathic procedures, the absence of mechanical hypersensitivity in P2X4KO male and female mice was also observed using the sciatic nerve ligature model (Figure S1C).

In addition to evaluating evoked pain using Von Frey procedure, we also assessed the capacity of neuropathic animals to use their hurt paw through the weight-bearing test, which measures the weight distribution of the hind paw (Figure 1A, right panel). To account for differences in body weight among mice, we calculated the ipsilateral/contralateral weight paw ratio. Our data revealed that both male and female WT and P2X4KI mice reduced the weight applied on the neuropathic hind paw, as indicated by a significant decrease in the ratio suggesting that mice applied less weight on the injured paw. P2X4KO animals did not present any alteration of the ratio when compared to the sham condition, indicating the use of both paws to ensure posture and walk and the absence of mechanical pain in these animals.

To confirm the involvement of P2X4 in mechanical hypersensitivity induced by SNI in WT and P2X4KI mice (Figures 1B and 1C) or in basal condition in P2X4KI mice (Figure 1C), we intraperitoneally injected the specific P2X4 antagonist 5-BDBD and measured the ipsilateral threshold and the weight hind paw repartition before and 2 h after injection in male and female WT mice (Figure 1B). While the antagonist did not alter the threshold in sham condition (Figure S2), 5-BDBD injection induced a significant reversion of the hypersensitivity induced by neuropathy in male and female WT mice 15 days after SNI. Using the weight-bearing test, we found similar results in male and female mice, with a significant increase in the paw ratio induced by 5-BDBD injection. Interestingly, P2X4 antagonist injection in P2X4KI mice of both sexes not only reversed the hypersensitivity induced by neuropathy (Figure 1C) but also the basal hypersensitivity of naive P2X4KI mice, restoring the mechanical threshold in males and females to levels close to those measured in WT mice (Figures 1C and S4).

The well-characterized P2X4-induced pain pathway has demonstrated that BDNF release from spinal microglia plays a crucial role in the development of tactile allodynia in male.^22^^,^^23^ Since our previous results demonstrated a pivotal role of P2X4 in neuropathic female, we examined whether BDNF is also involved in the mechanical hypersensitivity. For this, we administered Ana-12, a BDNF TrkB receptor antagonist, as we did previously for the P2X4 antagonist. Using electronic and manual Von Frey tests, we found that the Ana-12 injection reversed the hyperalgesia induced by SNI in both male and female WT and P2X4KI mice (Figures 1D and 1E), without any effect on sham nor vehicle-injected mice (Figures S2 and S3). Similar to the effect of 5-BDBD on P2X4KI mice, Ana-12 partially restored the basal mechanical threshold in males and females of P2X4KI to levels close to those measured in naive WT mice (Figure S4). Together, these results indicate that P2X4 receptors and BDNF are necessary to neuropathic pain development in both male and female mice.

### Neuropathic pain is dependent on microglial P2X4 independently of the sex

**Figure 2:**
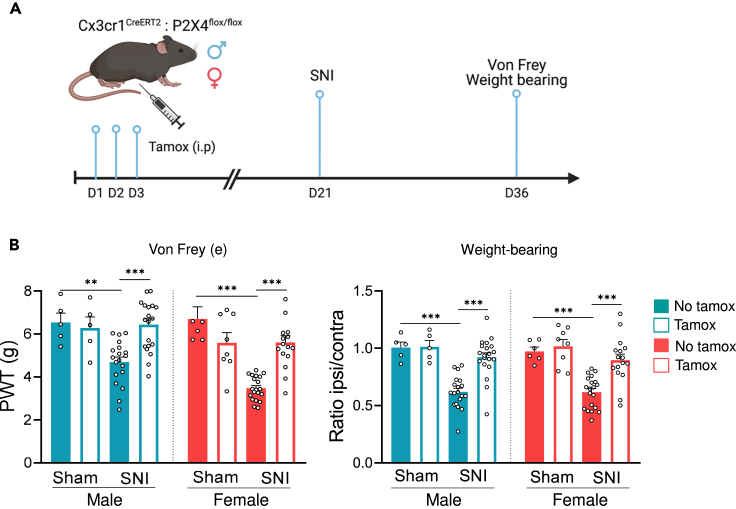
Specific deletion of P2rx4 in microglia reverses mechanical hypersensitivity in males and females (A) Schematic representation of the designed experiments with Cx3cr1^CreERT2^xP2X4^flox/flox^ mice (myeloid P2X4KO). (B) Paw withdrawal threshold (PWT) measured with the electronic Von Frey (left panel) and ipsilateral/contralateral ratio on the weight repartition using the weight-bearing test (right panel) in male and female myeloid P2X4KO mice. *P2rx4* deletion in microglia reverses mechanical sensitivity induced by SNI only in tamoxifen-injected mice. Data are represented as mean ± SEM. N = 6–20 mice. ∗∗p < 0.01, ∗∗∗p < 0.001, three-way ANOVA.

Although several studies, including our own, suggest a prominent role of microglial P2X4 in chronic neuropathic pain, other studies have described the expression of P2X4 in sensory and central neurons,^14^^,^^15^^,^^20^^,^^24^ raising the question of whether microglial or neuronal P2X4 is involved. To decipher which cell type contributes to tactile allodynia observed during neuropathy, we specifically deleted *p2rx4* in myeloid cells using tamoxifen-inducible Cx3cr1^CreERT2^:P2X4^flox/flox^ mice, in which P2X4 is specifically deleted in myeloid cells. Mice were injected or not with tamoxifen and SNI was performed 21 days later (Figure 2A). Interestingly, *p2rx4* deletion in microglia reversed the mechanical sensitivity in both male and female SNI mice using the Von Frey test, when compared to the sham condition (Figure 2B). Consistent with this result, the weight-bearing test revealed that *p2rx4* deletion increased the ipsilateral/contralateral ratio in both sexes.

**Figure 3:**
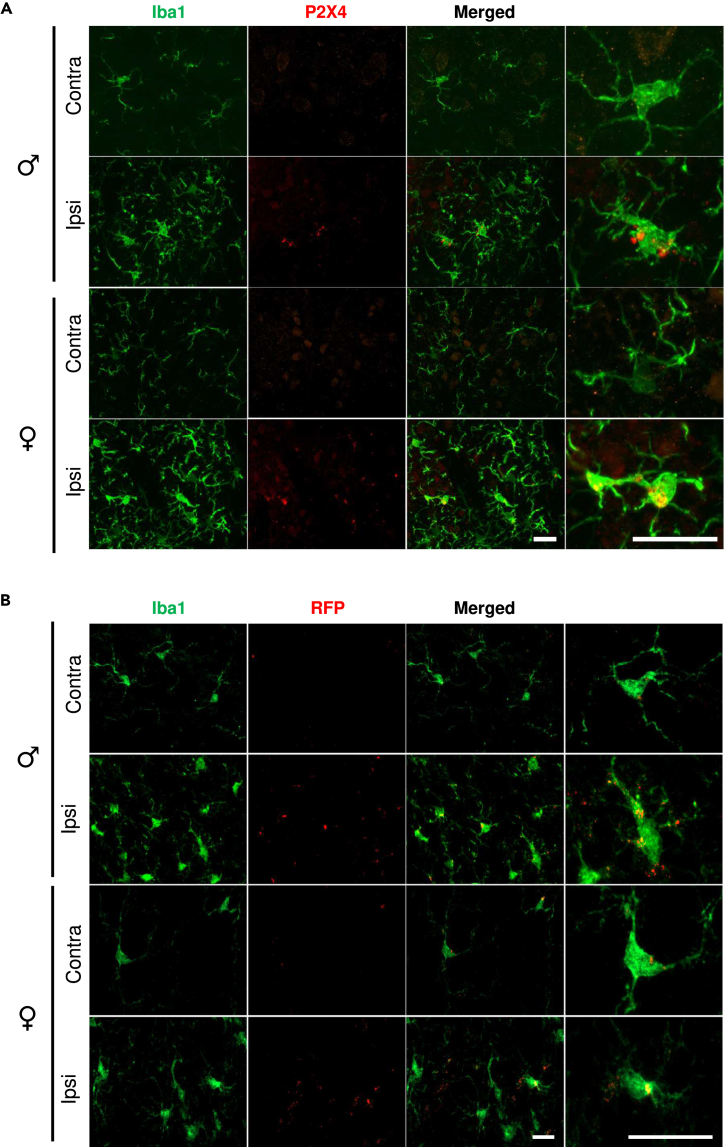
Similar P2X4 expression in male and female neuropathic mice (A) Representative images of P2X4 immunostaining using anti-P2X4 antibody of ipsilateral and contralateral side of the spinal cord of SNI mice. P2X4 (red) colocalized with Iba1+ cells (green) in the ipsilateral side of the neuropathic spinal cord of WT male and female mice, whereas no staining is observed in the contralateral side nor in sham condition. Scale bar 20 μm. (B) Representative images of P2X4mCherryIN staining (using anti-RFP antibody) in sham and SNI conditions in the ipsilateral and contralateral side of the spinal cord of P2X4KI male and female mice. Scale bars: 20 μm.

Finally, we confirmed that the increase in P2X4 expression occurs selectively in spinal microglia following SNI in both male and female WT and P2X4KI mice (Figure 3). Double immunostaining using either anti-P2X4 to detect P2X4 in WT mice (Figure 3A) or anti-RFP to detect P2X4mCherryIN expressed in P2X4KI mice (Figure 3B) with the microglial marker anti-Iba1 revealed that P2X4 is only expressed in Iba1-positive microglial cells in the ipsilateral dorsal horn of the lesion in both male and female WT and P2X4KI mice. No or very faint P2X4 staining was detected in the contralateral dorsal horn of the spinal cord.

Taken together, our results demonstrate that microglial P2X4 in the dorsal horn of the spinal cord plays a critical role in the mechanical hypersensitivity and is necessary for the development and maintenance of neuropathic mechanical hyperalgesia in both male and female mice.

### P2X4-independent morphological reactivity of spinal microglia in male and female mice during neuropathy

**Figure 4:**
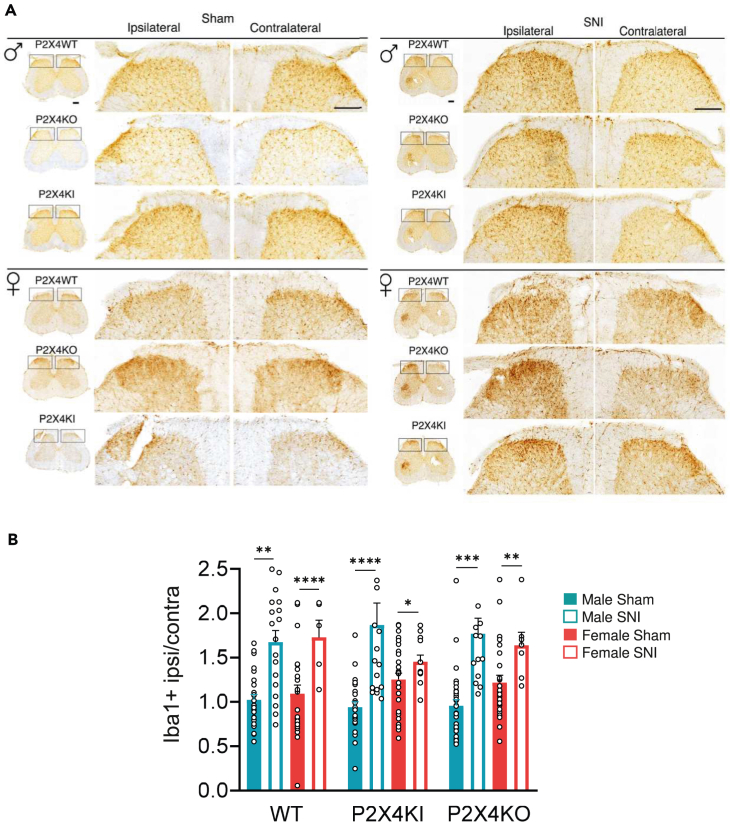
p2rx4 deletion does not affect microglial morphology in the dorsal horn of the spinal cord (A) Representative images of Iba1 staining in the ipsilateral and contralateral dorsal of the spinal cord from sham and SNI male and female mice. Scale bars: 200 μm. (B) Quantification of the area covered by microglia from A. A significant increase of the Iba1 area was observed in the ipsilateral side of the DH from the three genotypes. Data are represented as mean ± SEM. N = 3 mice per group. ∗p < 0.05, ∗∗p < 0.01, ∗∗∗p < 0.005, ∗∗∗∗p < 0.001, two-way ANOVA.

A hallmark of the neuropathic pain is the reactivity of the spinal microglial cells, characterized by proliferation and morphological modifications.^25^ We examined changes of microglia stained using anti-Iba1 antibodies by quantifying the positive stained area in the dorsal horn of the spinal cord of the different mice. As shown in Figure 4A, the increase of Iba1 staining revealed that SNI induced microglial reactivity in the ipsilateral side of the lesion compared to sham or contralateral side condition in WT, P2X4KI, as well as P2X4KO in mice of both sexes. The ipsilateral vs. contralateral ratio of Iba1-positive area in the dorsal horn quadrant revealed a significant increase of the surface occupied by microglia that was similar in the three genotypes in males and females (Figure 4B). These results indicate that SNI induces microglial reactivity in the spinal dorsal horn of both male and female mice that is independent of P2X4.

### Lymphocytes recruitment in the spinal cord is sex and P2X4 independent in neuropathy

**Figure 5:**
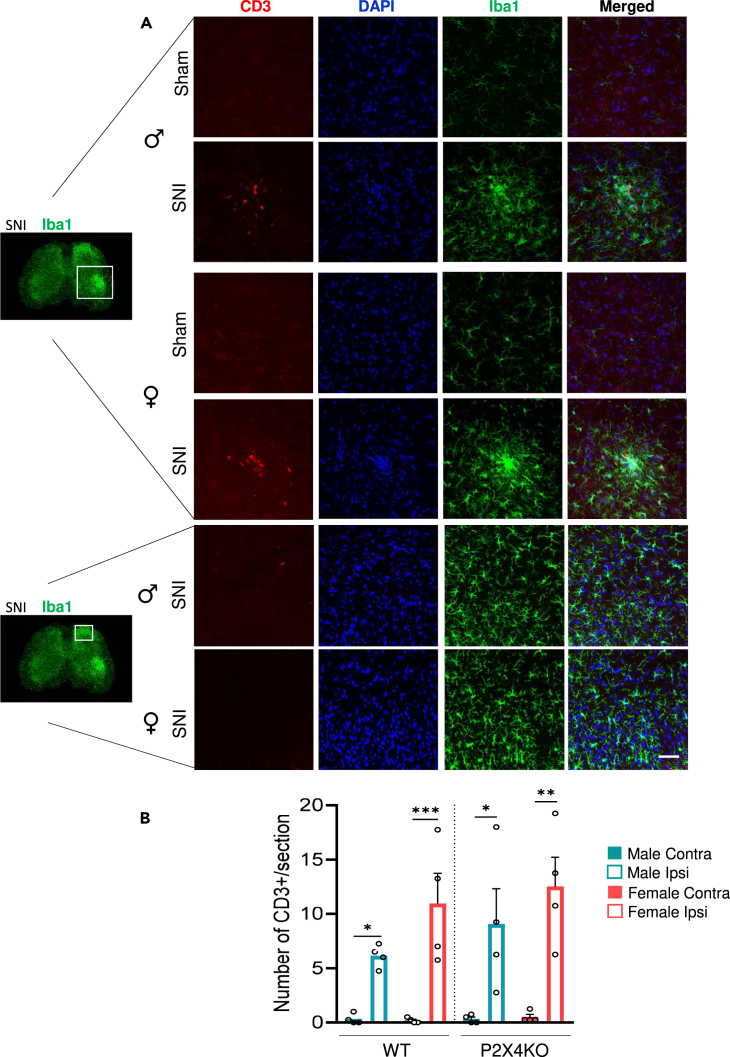
Neuropathy induced lymphocytes recruitment in the ventral DH of neuropathic mice (A) Representative images of CD3 (red) and Iba1 (green) immunohistochemistry from ventral and dorsal horn of the spinal cord from sham and SNI WT mice. Neuropathy induced CD3^+^-positive cells recruitment in the ventral horn of both male and female mice. No lymphocyte was observed in the dorsal horn of male or female neuropathic mice. (B) Quantification of the numbers of CD3^+^ cells in the ventral horn of sham and SNI WT and P2X4KO mice. SNI induced similar lymphocytes recruitment in male and female WT and P2X4KO mice. Data are represented as mean ± SEM. N = 4 mice per condition. ∗p < 0.05, ∗∗p < 0.01, ∗∗∗p < 0.005, two-way ANOVA.

Next, we investigated the recruitment of T lymphocytes in the neuropathic spinal cord, where their presence has been suggested to play an alternative role to microglial P2X4 in females.^9^ We examined and quantified the number of CD3-positive cells (CD3^+^ lymphocytes) using anti-CD3 antibody in sham and SNI WT as well as P2X4KO mice. Surprisingly, we did not find CD3^+^ cells in the dorsal horn of the spinal cord in sham nor in SNI mice. By contrast, CD3^+^ lymphocytes were observed in the ipsilateral ventral horn of SNI mice, predominantly around motoneurons area of both male and female WT and P2X4KO mice (Figure 5A). The quantification of CD3^+^ cell number in the ventral horn indicated a significant recruitment of lymphocytes in the two genotypes (Figure 5B), which was similar in both males and females.

### Increased dorsal horn neurons responsiveness to nociceptive inputs required microglial P2X4

**Figure 6:**
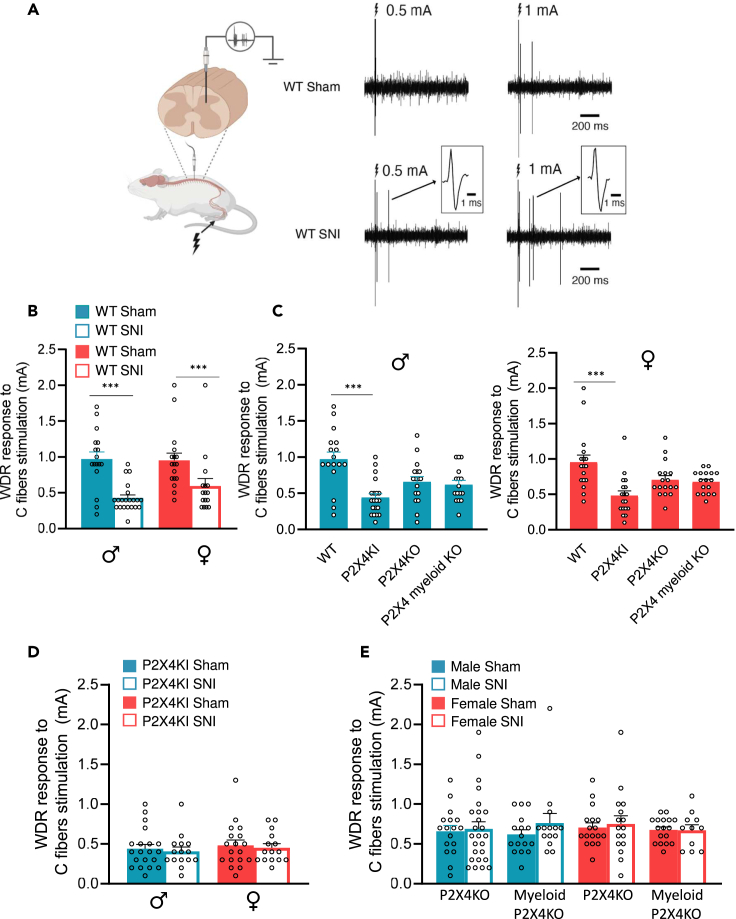
P2X4-dependent dorsal horn neuron hyperexcitability (A) *In vivo* single unit recordings of WDR neurons in response to C-fiber electrical skin stimulation. C-fiber threshold is the minimal intensity of the peripheral receptive field inducing a response of WDR neurons with a latency between 80 and 300 ms. Note that in SNI, a low intensity stimulation of C-fibers induces a WDR response not present in sham mice. Examples of action potentials recorded are also showed at higher timescale in insets. (B) SNI procedure induces a significant decrease of C-fiber threshold both in males (in blue) and females (in red). In males, minimal intensity stimulation to elicit WDR C-fiber component is significantly lowered in WT SNI mice (0.44 ± 0.05 vs. 0.97 ± 0.09, ∗∗∗p < 0.001). In females, minimal intensity stimulation to elicit WDR C-fiber component is significantly lowered in SNI mice (0.48 ± 0.06 vs. 0.95 ± 0.1, ∗∗∗p < 0.001, two-way ANOVA). (C) In P2X4KI mice, the C-threshold is significantly decreased as compared to WT and not significantly modified in P2X4KO mice (total or myeloid). This effect is similar in males (blue, right histograms) and females (red, left histograms). ∗∗∗p < 0.001, two-way ANOVA. (D) C-fiber threshold is not different from sham in P2X4KI after SNI procedure both in male (blue) and female (red). N = 15–20. (E) In both the general and myeloid-specific P2X4KO mice, the C-threshold response is equivalent in sham and SNI both in males (right, blue histograms) and females (left, red histograms). Data are represented as mean ± SEM.

Dorsal horn neuronal network integrates both non-nociceptive and nociceptive inputs. Particularly, deep dorsal horn neurons are projection neurons receiving both non-nociceptive and nociceptive inputs. We compared the response of wide dynamic range (WDR) neurons to nociceptive electrical skin stimulation in both male and female sham and SNI mice (WDR, see peristimulus histogram, Figure S5), focusing on their capacity to detect nociceptive C-fiber stimulations of their peripheral receptive field in anesthetized animals (Figure 6A). As already observed in rats,^26^ after SNI in WT mice, the threshold to exhibit a C-fiber response in WDR neurons was significantly lowered compared to sham animals in both males and females, indicating that SNI triggers dorsal horn neuron hyperexcitability in response to nociceptive stimuli (Figure 6B).

We then analyzed the consequences of the different P2X4 genotypes on dorsal horn excitability in WT and SNI male and female mice. In agreement with the mechanical hypersensitivity observed in naive male and female P2X4KI mice (Figures 1 and S1), we showed that the threshold to exhibit a C-fiber response in WDR neurons was significantly lowered in sham P2X4KI mice compared to sham WT mice in both male and female mice, indicating that increased cell surface P2X4 expression is sufficient to record dorsal horn hyperexcitability. By contrast, the threshold was not significantly different in P2X4KO global or in myeloid-specific KO mice compared to WT (Figure 6C).

Finally, we compared the WDR threshold to C-fiber simulation in sham and SNI conditions between P2X4KI and P2X4KO mice (Figures 6D and 6E). In P2X4KI mice, the WDR threshold to C-fiber stimulation was low and comparable between sham and SNI (Figure 6D). As expected, in general P2X4KO and myeloid cell-specific P2X4KO mice, the WDR threshold was not different between sham and SNI (Figure 6E). These results were comparable in male and female mice and strongly suggest that dorsal horn hyperexcitability to nociceptive inputs in SNI mice depends on P2X4 expression in microglial cells. Together, these results indicate that in our conditions, both male and female mice develop an identical P2X4-dependent hypersensitivity.

## Discussion

In this study, we demonstrated that cell surface expression of P2X4 (i) is sufficient to increase mechanical sensibility in both male and female naive mice and (ii) is indispensable for the development and maintenance of tactile allodynia and the associated hyperexcitability of spinal dorsal horn neurons in both male and female mice with neuropathy induced by the SNI model. Our findings are further supported by the absence of mechanical hyperalgesia and neuronal hyperexcitability in both male and female P2X4KO and myeloid cell-specific P2X4KO mice following SNI, as well as the similar decrease of mechanical hypersensitivity observed when using a specific P2X4 antagonist in neuropathic mice of both sexes. Collectively, these results strongly emphasize the crucial role of microglial P2X4 in neuropathic pain in both male and female mice.

### Cell surface increase of P2X4 expression is sufficient to trigger mechanical hypersensitivity

Importantly, we found that internalization-defective P2X4KI mice show mechanical hypersensitivity under basal condition compared to WT or P2X4KO mice. This hypersensitivity was similar in both male and female P2X4KI mice, demonstrating that increased cell surface P2X4 expression is sufficient to trigger mechanical hypersensitivity, independently of the sex. We suggest that this hyperexcitability originates from an increased activation of P2X4, which is induced by the basal release of ATP from neurons and astrocytes in the spinal cord.^27^^,^^28^ Interestingly, behavioral phenotyping of P2X4KI mice previously revealed that mice displayed no obvious abnormalities and were normal in body weight, postural or locomotor activities as well as for velocity, distance traveled in open field or exploratory time in maze,^20^ swimming performances, or postural scoring.^21^ Indeed, we never observed any signs of persistent pain in male or female P2X4KI mice, such as prostration, licking, or raising paw activities, indicating that the constitutive increase in cell surface P2X4 expression does not induce spontaneous and continuous pain perception in P2X4KI mice. However, it does generate mechanical hypersensitivity, as measured by the Von Frey test on both hind paws.

Moreover, when we performed the SNI procedure on these P2X4KI mice, we observed a reduction in the mechanical threshold of the ipsilateral side in both male and female mice, leading to the development of tactile allodynia similar to that observed in WT neuropathic mice. This finding is consistent with the increase in cell surface P2X4 expression in spinal microglia through *de novo* expression of microglial P2X4, as previously reported,^5^^,^^22^ associated with an increase of ATP secretion in the dorsal horn of the spinal cord.^29^ In P2X4KI mice, the *p2rx4* gene remains under the control of its own promoter,^20^ supporting the notion that this mechanism contributes to the observed increase in P2X4 expression.

### The microglial P2X4-dependent pathway is conserved in male and female neuropathic mice

Numerous studies have demonstrated that microglial cells play crucial roles in the development and the maintenance of neuropathic pain following peripheral nerve injury.^30^^,^^31^ Among the various immune functions, such as cytokines release, microglia induce neuronal hyperexcitability in the dorsal horn of the spinal cord and directly contribute to the synapses efficiency and central sensitization.^5^^,^^32^^,^^33^ In this model, *de novo* P2X4 receptor expression is induced in reactive microglia.^5^ Activation of cell surface P2X4 by ambient ATP originating from neurons and astrocytes triggers the release of BDNF by microglia, which is responsible for neuronal hyperexcitability in male neuropathy through its action on TrkB receptor.^22^^,^^23^^,^^29^ In addition, it has been shown that intrathecal injection of BDNF results in mechanical allodynia in naive male and female animals through decreased KCC2 expression.^11^ However, specific deletion of BDNF in microglia only prevents mechanical hypersensitivity in males, with no effect in females,^9^ indicating that the involvement of microglial BDNF in females remains controversial. Here, we demonstrate that pharmacological inhibition of TrkB during neuropathy reduces mechanical hypersensitivity to a similar extent in males and females of both WT and P2X4KI mice, strongly suggesting the conservation of the P2X4-BDNF-TrkB-KCC2 pathway in both sexes.

Morphological changes of microglia induced by neuropathy were similar in WT, P2X4KO, and P2X4KI male and female mice, suggesting that P2X4 is not involved in microglial reactivity during neuropathy. Our results are consistent with previous work showing no difference in proliferation or microglial morphology between males and females in spinal cord during chronic constriction injury.^34^ While microglial reactivity and pain phenotype are comparable in both sexes, a sexual dimorphism has been observed with genetic or pharmacological inhibition of P2X4 and its related pathway.^9^ However, P2X4 expression has been detected in male and female microglia in other pain models, such as herpetic pain^13^ or cancer pain.^12^ Our immunostaining results using two distinct antibodies (anti-P2X4 and anti-RFP) detecting, respectively, P2X4 in WT mice and P2X4mCherryIN in P2X4KI mice, revealed that SNI induces *de novo* P2X4 expression in spinal microglia in males but also in females of both WT and P2X4KI mice. The involvement of P2X4 in neuropathic pain in animals of both sexes is indeed confirmed (i) by the similar analgesic effect of the P2X4 antagonist 5-BDBD, which reduces the mechanical allodynia in both male and female WT and P2X4KI neuropathic mice, and (ii) by the absence of tactile allodynia following SNI in male and female P2X4KO mice. Furthermore, the absence of tactile allodynia was also observed in inducible myeloid-specific P2X4KO mice after SNI in both males and females. Although we cannot fully rule out the involvement of P2X4 expressed in macrophages, our data strongly suggest that microglial P2X4 is required not only for tactile allodynia in males, in agreement with extensive data,^9^^,^^22^ but also in females.

### P2X4 is required for spinal neuron hyperexcitability

Neuronal hyperexcitability in the dorsal horn of the spinal cord directly contributes to the development and maintenance of neuropathic pain following peripheral nerve injury.^35^ Here, we present evidence of the involvement of microglial P2X4 receptors in spinal neuron hyperexcitability during neuropathic conditions. This was demonstrated through *in vivo* recordings of the response of spinal WDR neurons to nociceptive electrical skin stimulation of their peripheral receptive field in the hind paw of anesthetized animals.^36^^,^^37^^,^^38^ Associated with the mechanical hypersensitivity observed in male and female naive P2X4KI mice (Figures 1 and S1), we demonstrate a significant decrease in the threshold to elicit a C-fiber response in WDR neurons of sham P2X4KI mice compared to sham WT mice in both males and females. This is in accordance with previous studies in both rats and mice showing increased WDR hyperexcitability in pathological model of persistent pain.^26^^,^^39^ This hyperexcitability was consistent across both male and female mice. By contrast, no changes in the WDR threshold following C-fiber stimulation were observed after SNI in P2X4KO mice or myeloid P2X4KO mice of both sexes, demonstrating the involvement of P2X4 in myeloid cells in dorsal horn neuron hyperexcitability. Furthermore, we found no changes in the WDR threshold in P2X4KI mice after SNI. This result appears contradictory to the enhanced mechanical hypersensitivity observed in P2X4KI mice after SNI, but several hypotheses can be proposed. It is possible that C-fibers are not the only fibers conducting nociceptive inputs, and alterations in Aδ fibers^40^ or non-nociceptive Aβ fibers^41^ could contribute to the observed effects. In addition, we cannot exclude that other projection neurons, such as nociceptive-specific neurons of lamina I, are also altered and contribute to the increased mechanical hypersensitivity observed in P2X4KI mice after SNI.^42^ Nevertheless, the present results show a spinal hyperexcitability of WDR neurons after SNI in both males and females. This hyperexcitability is absent when myeloid P2X4 is knocked out. Moreover, the upregulation of cell surface P2X4, which likely results in increased P2X4 activity, is sufficient to induce WDR hyperexcitability. Finally, global and myeloid-specific P2X4 knockout is sufficient to eliminate SNI-induced spinal neuron hyperexcitability in both male and female mice, further highlighting the prominent role of myeloid P2X4 in the generation of spinal plasticity underlying nociceptive hypersensitivity.

### Similar lymphocyte infiltration induced by neuropathy

Although our results strongly suggest that microglial P2X4 is essential for the development of tactile allodynia associated with dorsal horn neurons hyperexcitability in both males and females under neuropathic conditions, the involvement of T cells has been proposed specifically in female,^9^ in which infiltration of T lymphocyte in the spinal cord could actively suppress microglial activation.^9^^,^^11^ However, in line with previous data,^43^ we did not detect lymphocyte infiltration in the ipsilateral dorsal horn of both male and female mice in our neuropathic pain model, despite observing strong microglial reactivity in this region. Even if we cannot rule out that T cells in the ventral horn contribute to the maintenance of chronic pain, the absence of preferential expansion of T cells in female and the absence in the dorsal horn of the spinal cord in both sexes suggest that spinal infiltrating T cells are not essential for the development and maintenance of mechanical allodynia induced by the SNI model. Moreover, the equivalent infiltration measured in P2X4KO mice and the absence of P2X4 detection in spinal cord ventral horn (data not shown) suggests that this specific spinal lymphocyte infiltration is independent of microglial P2X4 pathway, supporting the previous hypothesis. Several studies have, however, reported that lymphocytes are necessary for the development and chronicity of neuropathic pain.^44^ In particular, an important infiltration of pro-inflammatory CD4^+^ lymphocytes occurs in the leptomeninges of the dorsal root ganglia in neuropathic pain model or in spinal meninges in CSF1 neuropathy-mimicking model, which may interact with microglia to modulate mechanical hypersensitivity.^43^^,^^45^ Further investigations are needed to characterize meningeal lymphocytes in both sexes and determine potential sexual dimorphism.

### Difference in murine breeding could explain sexual dimorphism

The discrepancies between the putative sexual dimorphism of the P2X4-dependent pathway in neuropathic could arise from differences in the tested murine lineages. Indeed, several studies have demonstrated that differences in pain responses can be attributed to genetic variations among mouse lineages.^46^ Moreover, anti-nociception mechanisms are also dependent of sex in a given lineage.^47^^,^^48^ It is conceivable that breeding conditions of the mice could influence their physiological and pathophysiological responses. Indeed, the environment has been shown to be crucial not only for the physiological parameters but also for pathogenesis of chronic neuropathic pain and other chronic diseases.^46^ Male and female animals may respond differently to environmental modifications, with females often exhibiting a more pronounced sensitivity.^46^ The environment of the mice depends mainly on the health status of the animal facilities. Our mice were bred in two distinct specific opportunist and pathogen-free barrier facilities located in different cities and were housed during *in vivo* experiments in a separate conventional facility with sanitary status close to specific pathogen-free status. Therefore, our animals were not exposed to microorganisms that might be present in other animal facilities and could potentially modify their immune responses.^48^ The effects of environmental modifications have been studied and have revealed significant remodeling of the innate and adaptive immune systems,^49^ in line with data showing that microbiota influence inflammatory responses, neuronal excitability, and microglial activity in a model of neuropathy.^50^ Moreover, it has been demonstrated that the absence of microbiota induces an alteration of the immune phenotype of microglial cells and that the presence of complex microbiota is necessary for the maintenance of functional microglia.^51^ This emphasizes the potential fundamental role of the microbiota in brain immunity. Comparisons of health status of animals between studies in the future may provide crucial information.

In conclusion, our findings indicate that cell surface P2X4 expression is sufficient to induce mechanical hypersensitivity and increase spinal neuron excitability in naive P2X4KI mice of both sexes. Furthermore, the upregulation of P2X4 in spinal microglia of both male and female mice is crucial for the development of tactile allodynia and neuronal hyperexcitability in neuropathic pain model.

## STAR★Methods

### Key resources table

**Table:** 

REAGENT or RESOURCE	SOURCE	IDENTIFIER
**Antibodies**		

Nodu-A246 rat monoclonal against native extracellular domain of mouse P2X4 antibody	Bertin et al.^20^^,^^21^	N/A
Rabbit anti-P2X4	Alomone Labs	Cat# APR-002
Rabbit anti-RFP	MBL	Cat#PM005
Rat anti-CD3	BD Biosciences	Cat#555273
Rabbit anti-Iba1	Abcam	Cat#AB178846
Goat Anti-Iba1	Abcam	Cat#ab5076
Rabbit polyclonal Iba1	Wako	Cat#019-19741
Donkey anti-rat A594	Jackson Immunoresearch	Cat#712-586-150
Anti-rabbit HRP	DAKO	Cat#K400311
Donkey anti-rabbit CF488A	Sigma	Cat#SAB4600036

**Chemicals, peptides, and recombinant proteins**		

Formalin solution neutral buffered 10%, 4% PFA	Sigma-Aldrich	Cat#HT501128
5-BDBD	Tocris	Cat#3579
ANA-12	Sigma-Aldrich	Cat#SML0209
DMSO	Sigma-Aldrich	Cat#D2650
Tamoxifen	Sigma-Aldrich	Cat#T5648
corn oil	Sigma-Aldrich	Cat#C8267
Kit revelation DAB (IMN)	DAKO	Cat#K3468

**Experimental models: Organisms/strains**		

P2X4KI mice = P2X4mCherryIN mice	Bertin et al.^20^^,^^21^	N/A
P2X4KO mice	Sim et al.^15^	N/A
C57BL/6J mice	Jackson Laboratory	Jax #000664
CX3CR1CreERT2	Yona et al.^52^	N/A
P2X4KO^flox/flox^	Ozaki et al.^53^	N/A

**Software and algorithms**		

ImageJ	National Institutes of Health, USA	https://imagej.nih.gov/ij/
Prism	Graphpad	www.graphpad.com

**Others**		

Static weight-bearing test	Bioseb	BIO-SWB-TOUCH-M
Electronic Von Frey	IITC Life Science	Anesthesiometer
Manual Von Frey	Ugo Basile	37450–275

### Resource availability

#### Lead contact

Further information and requests for resources and reagents should be directed to and will be fulfilled by the lead contact, Lauriane Ulmann (lauriane.ulmann@igf.cnrs.fr)

#### Materials availability

This study did not generate new unique reagents.

### Experimental model and study participant details

#### Animals

Female and male mice from 6 to 10 weeks old were housed under a standard 12 h light/dark cycle with food and water available *ad libitum*. All the animals used were in C57BL/6J background. Mice carrying a null mutation for P2X4 gene (P2X4KO) have been described previously.^15^ Cx3cr1^CreERT2^ mice were obtained from Steffen Jung’s lab.^52^ P2X4^flox/flox^ mice were obtained from Rieko Muramatsu’s Lab.^53^ From these mice, we crossed Cx3cr1^CreERT2/+^ x P2X4KO^flox/flox^ to obtain myeloid cell-specific P2X4KO mice. Internalization-defective P2X4mCherryIN mice (P2X4KI) have been previously described.^20^^,^^21^ All the mice were bred either in the SPF animal facility of the Institute for Functional Genomic (IGF, Montpellier, France; Agreement from the Ministry of Agriculture N° D34-172-13) or in the Pole In Vivo-Expe and EOPS production facilities of the Center Broca-Nouvelle Aquitaine of Bordeaux (Agreement from the Ministry of Agriculture are respectively N° A33-063-940 and N° A33-063-941).

#### Study approval

All experimental procedures complied with official European guidelines for the care and use of laboratory animals (Directive 2010/63/UE) and were approved by the ethical committee of Bordeaux (CEEA50) and Montpellier (CEEA36) and the French Ministry of Agriculture APAFIS#22741–2019102914341098 v4) and APAFIS#21135-2019061914043519v3).

### Method details

#### Spared nerve injury (SNI)

Mice were randomly assigned to SNI or sham group. Animals were anesthetized under 3% isoflurane/oxygen, the left leg sciatic nerve were exposed and the peroneal and tibial branches were ligated and transected. Skin was closed with silk sutures. Identical procedure was performed in sham operated mice except the nerve ligation and transection. The mice were isolated until they had fully recovered from the surgery before being reintroduced with their littermate.

#### Partial nerve ligation (SNL)

Partial nerve ligation model was performed as previously described.^22^ Briefly, mice were anesthetized under 3% isoflurane/oxygen and the left leg sciatic nerve were exposed. A suture was passed through the dorsal third of the nerve and tied tightly. Skin was closed with silk sutures. Identical procedure was performed in sham operated mice except the nerve ligation. The mice were isolated until they had fully recovered from the surgery before being reintroduced with their littermate.

#### Drugs

P2X4 antagonist 5-BDBD (3579, Tocris) was dissolved in DMSO (D2650, Sigma) and intraperitoneally injected (28 mg/kg). TrkB antagonist Ana-12 (SML0209, Sigma), was dissolved in DMSO and intraperitoneally injected (1 mg/kg). Drug were aliquoted and stored at −20°C. Control mice only received DMSO. Drugs were injected 15 days after the surgery and behaviors were performed before and 2h after the injection. To induce recombination in Cx3cr1^CreERT2^ mice line, tamoxifen (T5648, Sigma) was dissolved at 10 mg/ml in a warm solution containing corn oil (C8267, Sigma) and 2.5% of absolute ethanol. Animals were injected with 1 mg of tamoxifen intraperitoneally one time a day during 3 days. Surgery was performed 21 days after the last injection.^54^

#### Mechanical hyperalgesia test

To test mechanical hyperalgesia, mice were habituated in Plexiglas compartments (5 cm × 10 cm x 14 cm) on a perforated metal floor for at least 45 min before the test. The hind paws withdrawal thresholds were measured on the different mouse lines in two distinct laboratories (Montpellier and Bordeaux) using electronic and manual Von Frey (Anesthesiometer, IITC Life Science, USA). For electronic Von Frey, calibrated tips were applied at the lateral plantar surface of the hind paw and response was recorded. Three measures per hind paw were recorded and averaged. For manual Von Frey (Ugo Basile, Italy), we calibrated Von Frey filament (in grams) on the plantar surface of the hind paws of the mice. Three to five measurements were made for each paw, with an interval of 30 s between each. The smallest grams of the filament at which the mouse withdrew its paw was taken as the mechanical pain threshold.^55^

#### Weight distribution changes test

Change in the hind paw weight distribution were assessed using the static weight-bearing test (WB), an incapacitance apparatus (BIO-SWB-TOUCH-M, Bioseb, France). Mice were place in a Plexiglas box with an inclined plane, which permit to maintain animals in position. The weight applied by hind paws is measured during 10s. A mean of the weight over 10 s is recorded. Results were presented as ratio contralateral over ipsilateral to avoid influence of the animal body weight on the measurement.

#### Tissue preparation

Mice were anesthetized with intraperitoneal injection of 200μL of Euthasol Vet diluted 1/7 in PBS and perfused intracardiacally with 20 mL PBS. Spinal cords were harvested and placed overnight in 2 or 4% paraformaldehyde. The contralateral side of the lesion was recognized by a scalpel’s notch. The L1 to S1 segments were cut with a vibratome (VT1000S, Leica) into 40 μm sections and stored in PBS +0.1% sodium azide at 4°C. When indicated, lumbar segments were cryoprotected in a solution of 20% sucrose within 0.1M PBS overnight and stored at −80°C. Using cryostat (Leica), series of 50 μm-thin coronal slices of the spinal cord were obtained.

#### Immunohistochemistry

The sections were washed with PBS. Non-specific sites were saturated with 10% donkey serum (S30, Millipore) diluted in PBS +0.3% Triton X-100 (Sigma, X100) for 1h at room temperature. Primary antibodies (rat anti-P2X4, 1:500, Dr Koch-Nolte, University of Hamburg-Eppendorf; rat anti-CD3, 1:500, 555273, BD Biosciences; rabbit anti-Iba1, 1:2000, AB178846, Abcam, rabbit anti-RFP, 1:500, PM005, MBL) were incubated overnight at 4°C in DPBS +0.3% Triton X-100. After three 15 min rinses in PBS, the sections were incubated for 2h at room temperature with the corresponding secondary antibody (donkey anti-rat A594, 1:1000, 712-586-150, Jackson Immunoresearch; donkey anti-rabbit CF488A, 1:1000, SAB4600036, Sigma) diluted in PBS +0.3% Triton X-100. The sections were rinsed with PBS 3 × 10 min and then mounted between slide and coverslip with mounting medium (Dako, S3023). L4 to L6 sections were observed using a fluorescence microscope (Axioimager 2, Zeiss) with an apotome grid module (Apotome 3, Zeiss) equipped with a digital camera (cool-snap HQ, PhotoMetrics) or a fluorescence confocal microscope (SP8-UV, Leica).

#### Lymphocytes quantification

CD3^+^ cells were quantified in all the sections of the L4, L5 and L6 spinal cord under fluorescence microscope in the 4 quarters of the spinal cord (dorsal/ventral and ipsilateral/contralateral). Results were expressed as the mean of the cell count.

#### Iba1 quantification

In order to assess the effect of the spared nerve injury on microglial surface occupancy in the spinal dorsal horn (SDH), Iba1 immunochemistry was performed. Lumbar sections of the spinal cord were selected and were incubated with rabbit polyclonal Iba1 antibody (1:2000, Wako, #019–19741) overnight night at room temperature. The slices were revealed the next day with the corresponding peroxidase EnVision secondary antibody (anti-rabbit, DAKO, K400311), followed by 3,3′-diaminobenzidine (DAB) visualization. Lumbar spinal cord sections were then mounted on gelatin-coated slides, dehydrated and cover-slipped. The slides were scanned using 3DHistech Pannoramic scan II slide scanner and images were analyzed using ImageJ software. Surface occupancy of the Iba-1 labeling was assessed in the ipsilateral and contralateral side to the lesion dorsal horns using a macro written in IMN lab by T. Dhellemmes. The ratio of the data obtained in the ipsilateral dorsal horn compared to the contralateral one was calculated for each section.

#### *In vivo* extracellular recordings

Mice were anesthetized with urethane 20% (1.5 g/kg) and placed on a stereotaxic frame (Unimécanique, Asnières, France). A laminectomy was performed on lumbar vertebrae L1–L3 and segments L4–L5 of the spinal cord were exposed. Extracellular recordings of wide dynamic range (WDR) dorsal horn neurons were made with borosilicate glass capillaries (2 M*Ω*, filled with NaCl 684 mM) (Harvard Apparatus, Cambridge, MA, USA). The criterion for the selection of a neuron was the presence of an A-fiber-evoked response (0–80 ms) followed by a C fiber-evoked response (80–300 ms) to electrical stimulation of the ipsilateral paw with subcutaneous bipolar electrodes connected to a stimulator and placed in the center of the recorded neuron receptive field. Threshold for C-fiber evoked response was evaluated by progressive increase in electrical stimulation intensity until the appearance of a C-fiber component.^36^

All measured are expressed as mean ± SEM. WDR threshold in each experimental group was compared using two-way ANOVA followed with Tukey’s post hoc A p value < 0.05 was considered significant.

### Quantification and statistical analysis

Data are represented as mean ± SEM and were analyzed with GraphPad Prism 8. Groups were compared using two-way ANOVA (post hoc with FDR correction) or a three-way ANOVA. The significance level refers to ∗p < 0.05, ∗∗p < 0.01, ∗∗∗p < 0.001, ∗∗∗∗p < 0.0001, ns 0.05 p < 0.05.

## Data Availability

This paper does not report original code. The sources of the datasets supporting the current study are presented in the “key resources table” and “STAR methods” sections. Any additional information required to reanalyze the data reported in this paper is available from the lead contact upon request.
