# Supplementary material for: Microglial P2X4 receptors are essential for spinal neurons hyperexcitability and tactile allodynia in male and female neuropathic mice
Source: iScience. 2023 Oct 2;26(11):108110. doi: 10.1016/j.isci.2023.108110 (PMC10583052; doi:10.1016/j.isci.2023.108110)
Supplement: Document S1. Figures S1–S5 [file mmc1.pdf]

iScience, Volume *26*

Supplemental information

Microglial P2X4 receptors are essential for spinal neurons hyperexcitability and tactile allodynia in male and female neuropathic mice

Damien Gilabert, Alexia Duveau, Sara Carracedo, Nathalie Linck, Adeline Langla, Rieko Muramatsu, Friedrich Koch-Nolte, François Rassendren, Thomas Grutter, Pascal Fossat, Eric Boué-Grabot, and Lauriane Ulmann

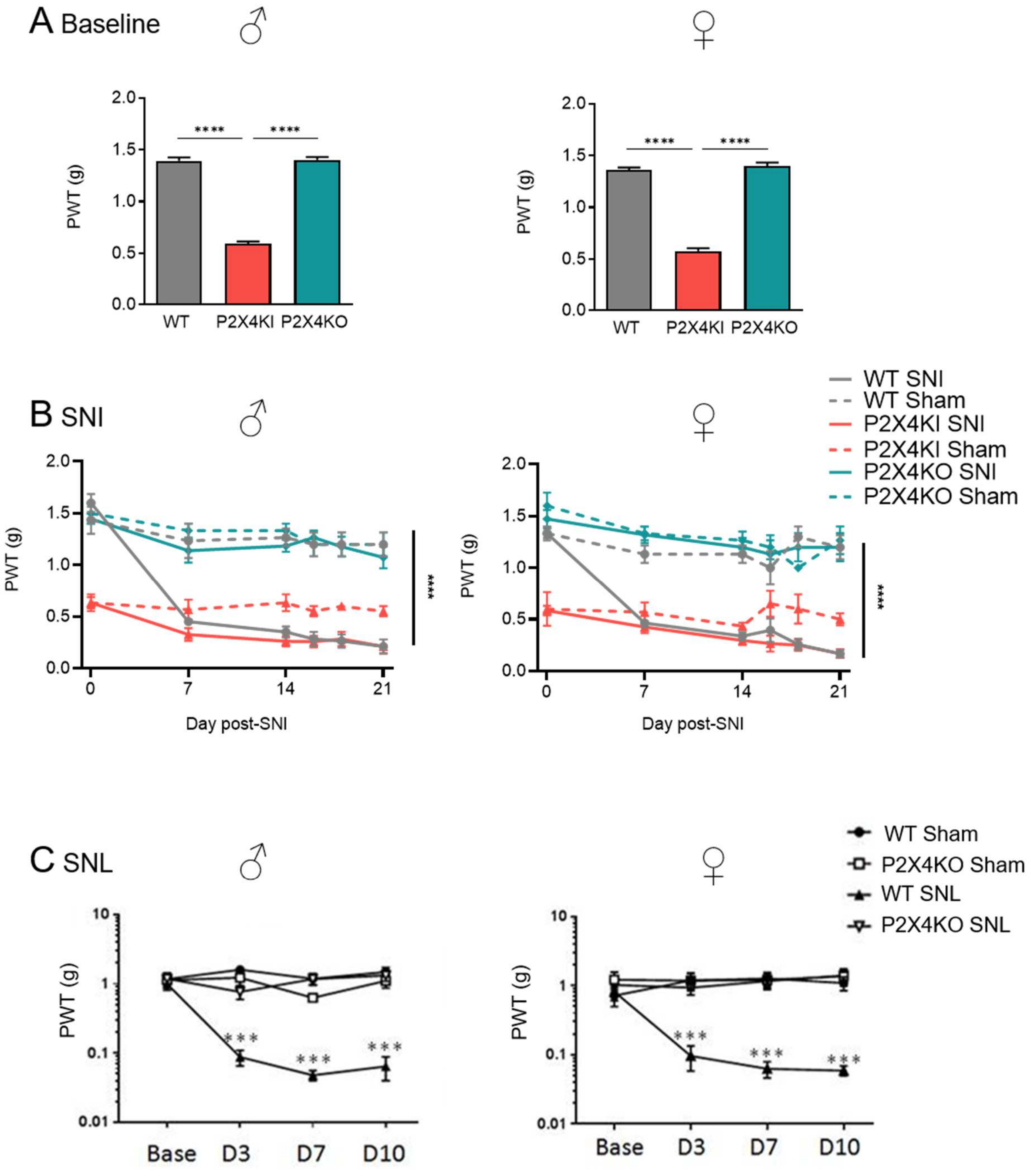

Figure S1. Increased P2X4 is sufficient to induce mechanical neuropathic pain in both male and female mice, related to Figure 1. (A) Manual Von Frey for the assessment of mechanical sensitivity of untreated male and female WT, P2X4KI and P2X4KO mice. P2X4KI mice present spontaneous hypersensitivity when compared to WT or P2X4KO mice. N= 60-62 mice per condition, One way ANOVA, **** $p<0.001$. (B) The tactile allodynia measured with manual Von Frey test developed from day 3, and at least until 21 days after surgery similarly for WT and P2X4KI mice of both sexes. The threshold of mechanical sensitivity was unchanged by SNI in P2X4KO male and female mice. N= 6-12 mice per condition, three way ANOVA, **** $p<0.001$. (C) Tactile allodynia from baseline to 10 days after sciatic nerve ligature in male and female WT and P2X4KO mice. P2X4KO mice do not develop mechanical hypersensitivity. N=8 mice per condition, Three way ANOVA, *** $p<0.005$.

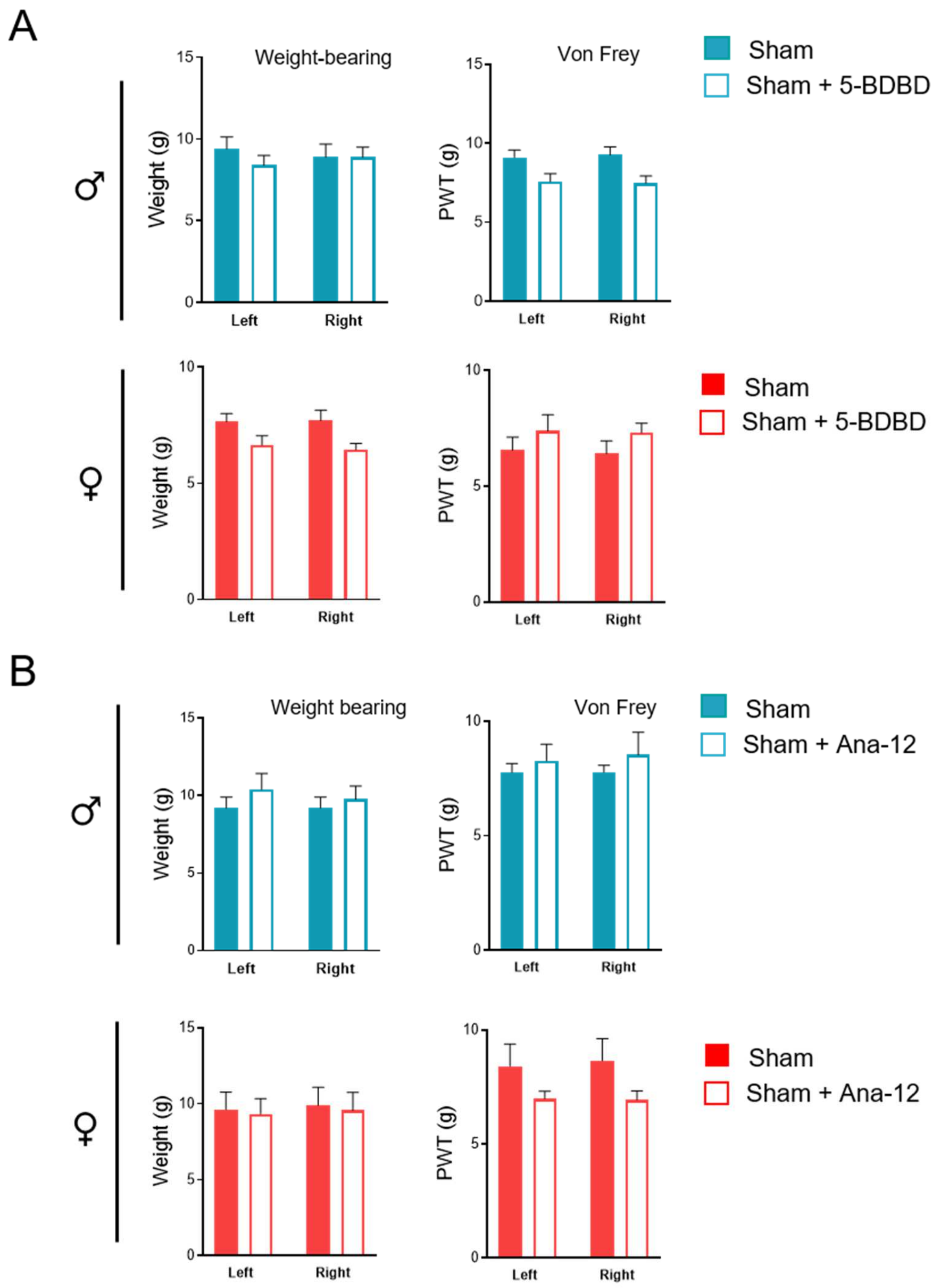

Figure S2. 5-BDBD and Ana-12 antagonists do not alter the mechanical threshold in sham condition, related to Figure 1B, C, D, E. (A) Weight-bearing tests (left panel) and manual Von Frey (right panel) for the assessment of mechanical sensitivity of male (top) and female (bottom) WT sham animals 2h after the P2X4 antagonist 5-BDBD (28 mg/kg,) i.p. injection. (B) Weight-bearing tests (left panel) and manual Von Frey (right panel) for the assessment of mechanical sensitivity of male (top) and female (bottom) WT sham animals 2h after the TrkB antagonist Ana-12 (1mg/kg) i.p. injection. Drugs has no effect on mechanical sensitivity in sham animals. Data are represented as mean ± SEM. N= 4 mice per condition, two-way ANOVA.

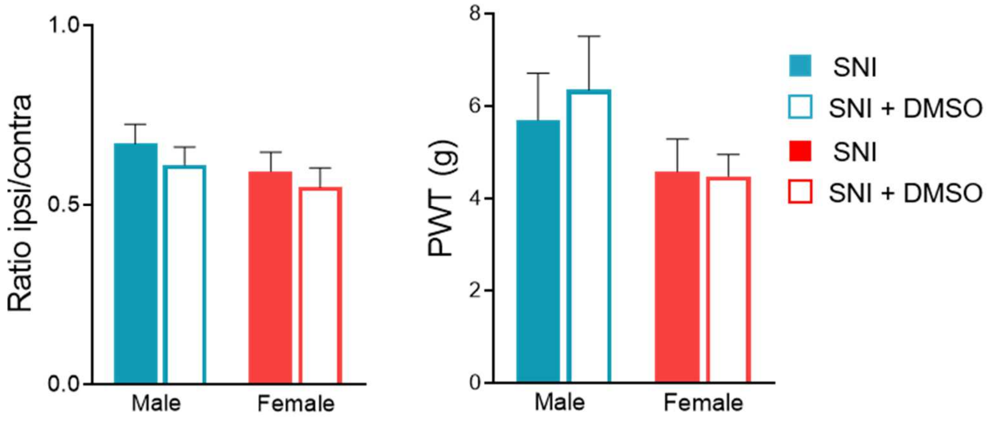

Figure S3. DMSO did not alter the mechanical threshold in sham condition, related to Figure 1B, C, D, E. Weight-bearing tests (left panel) and manual Von Frey (right panel) for the assessment of mechanical sensitivity of male (blue bars) and female (red bars) WT SNI animals 2h after DMSO i.p. injection. DMSO has no effect on mechanical sensitivity in SNI animals. Data are represented as mean ± SEM. N = 6-7 mice per condition, two-way ANOVA.

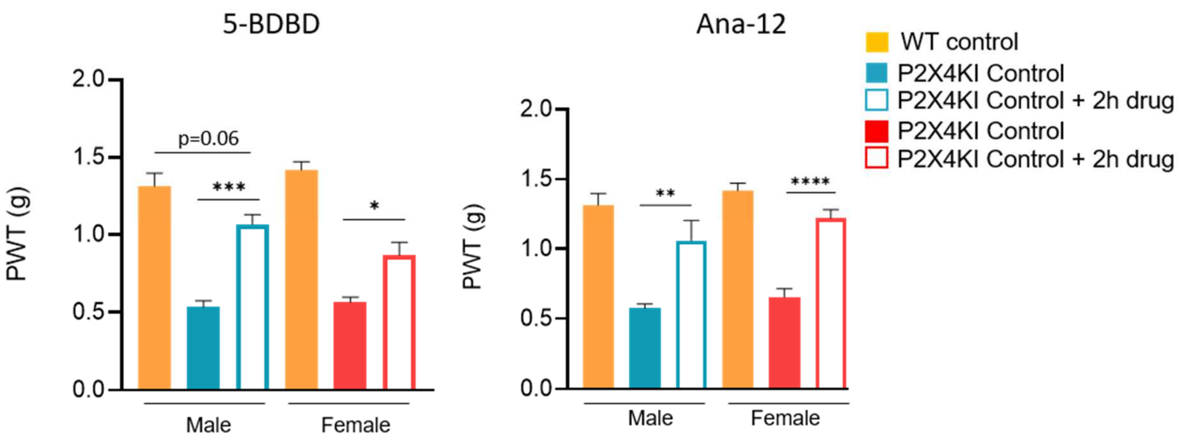

Figure S4. 5-BDBD and Ana-12 partially restored the basal mechanical threshold in males and females of P2X4KI, related to Figure 1D, E. Manual Von Frey for the assessment of mechanical sensitivity of control male (blue bars) and female P2X4KI mice (red bars) before and 2h after i.p. injection of either the P2X4 antagonist 5-BDBD (28 mg/kg,) (left panel) or the TrkB antagonist Ana-12 (1mg/kg) i.p. injection (right panel). P2X4 and TrkB inhibition reduce the hypersensitivity display by naïve P2X4KI mice to levels close to those measured in WT mice (orange bars). Data are represented as mean ± SEM. N= 6-12 mice per condition, * $p<0.05$, ** $p<0.01$, *** $p<0.005$, **** $p<0.001$, Student paired t-test.

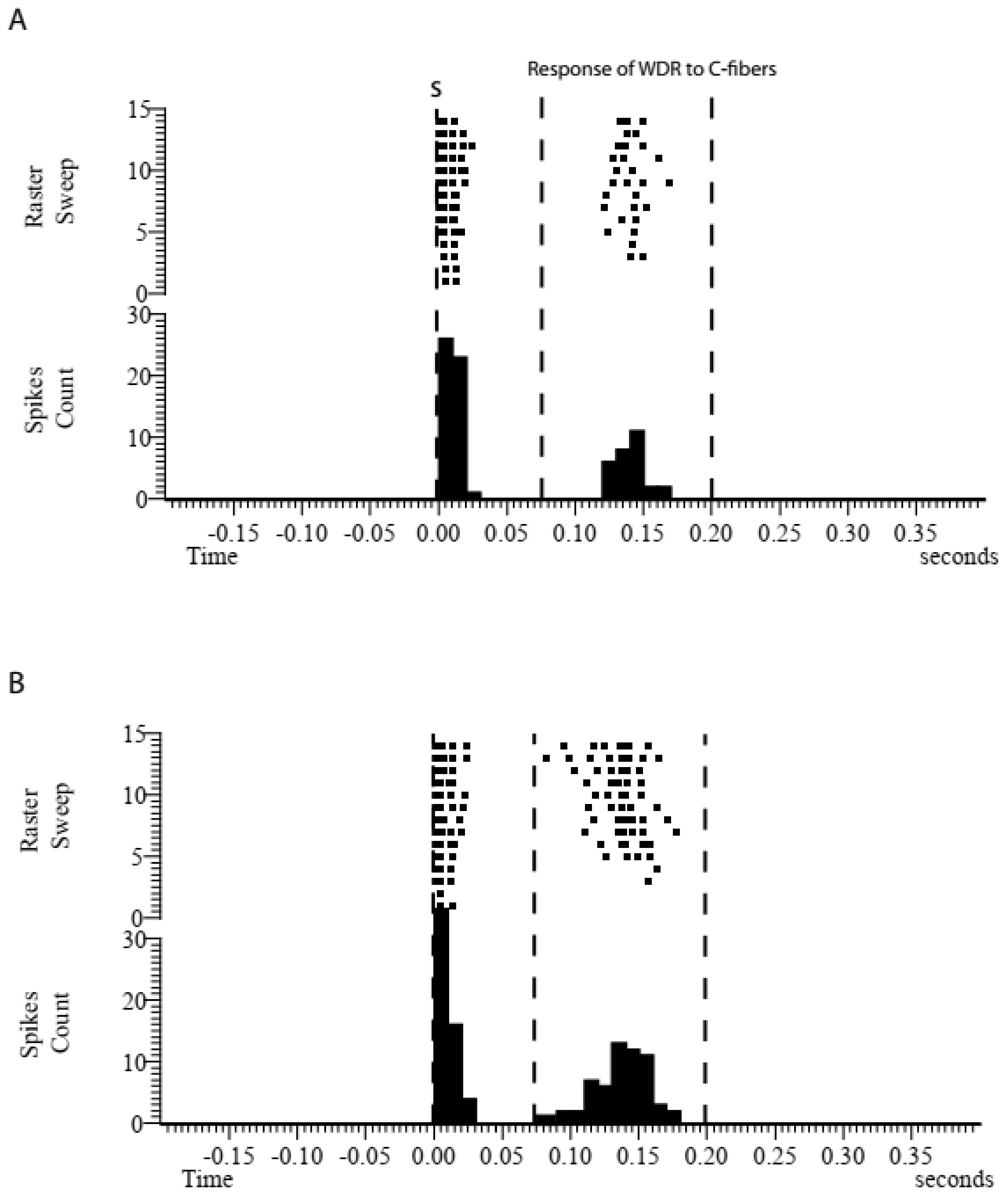

Figure S5. Peristimulus histogram (PSTH) of WDR neuron, related to Figure 6. Peristimulus histogram (PSTH) of WDR neuron recorded in WT (A) and P2X4KI mice (B). The response of WDR to C-fiber stimulation appears with a delay from the electric stimulation (s) (usually between 80 and 200 ms). The PSTH in P2X4KI show an increase in the number of C-spikes and enlargement of the WDR response to C-spikes. Data are represented as mean ± SEM.
